# Identifying Active Ingredients that Cause Change in Digital Parent Training Programs for Child Behavior Problems: A Qualitative Exploration

**DOI:** 10.1007/s10578-024-01803-1

**Published:** 2024-12-14

**Authors:** Chen R. Saar, Or Brandes, Amit Baumel

**Affiliations:** Department of Community Mental Health, University of Haifa, Abba Khoushy Ave 199, Haifa, 3498838 Israel

**Keywords:** Active ingredients, Digital, Parent training, Persuasive, Qualitative

## Abstract

Digital parent training programs (DPTs) aimed at treating child behavior problems have shown efficacy in a number of trials, but less is known about the active ingredients parents find helpful while using DPTs. We analyzed data from users of self-guided DPTs within a randomized controlled trial setting: a standard program (DPT-STD) and an enhanced program (DPT-TP). Thematic analysis of interviews (*n* = 16) reveals that users of both programs endorsed the “content”, “content presentation”, “accessibility”, and “therapeutic context” as beneficial. However, only DPT-TP users identified the “therapeutic persuasiveness” as helpful, attributing this to features embedded exclusively in the enhanced program, including call-to-action reminders and assessment-based feedback. Findings were reinforced by the analysis of responses to open-ended questions from a larger sample of users (*n* = 31 DPT-STD users and *n* = 34 DPT-TP users). These findings underscore the importance of utilizing features that help parents make positive changes in their home.

## Introduction

Behavioral parent training programs have gained widespread recognition for their effectiveness in addressing disruptive child behavior [40, 46, 54]. Through engagement with these programs, parents acquire both the knowledge and practical tools to enable them to observe and reform their responses to their child, thereby strengthening the appearance of the child’s positive behaviors and reducing problem behaviors.

Self-guided digital parent training programs (DPTs) were developed to overcome a treatment gap among parents of young children with problem behaviors who do not receive evidence-based treatment due to barriers such as cost, limited access to in-person treatment, stigma, or limited time [41]. Although there is a growing body of research on the effectiveness of DPTs for both parents and children (e.g., Baumel et al., [9, 12, 14, 27, 66], fewer studies have investigated what makes these programs effective, that is, what active ingredients within the program help parents to engage in positive behavior change [25, 35].

From a behavior change perspective, active ingredients of a program are the program-specific elements that promote behavior change [1, 37, 52, 64]. These components can be found within the content of the intervention (i.e., implementation of therapeutic principles, learning tools), the methods of delivery (i.e., text, videos), or the design features intended to encourage behavior change (i.e., reminders, feedback). For instance, sending automatic timely reminders to parents to practice quality time with their child along with a few ideas for mutual activities might improve the chance of parents accomplishing this desirable task between two successive parent training sessions [7]. The optimization of these aspects promotes the creation of more parsimonious interventions that ideally require users to invest less effort and resources [45, 50]. This iterative and ongoing process is invaluable for both the interventions’ developers and clinicians who are aiming to enhance the efficacy of parent training sessions.

There is a slowly growing body of literature on the active ingredients of parent training programs [5, 69]. Among the active ingredients identified in these studies are content-related components, such as teaching parents problem-solving and positive reinforcement, and setting-related components, such as adding home visits to the parent training intervention. Studies have also looked at specific techniques found to be helpful for children with severely challenging behavior, such as attunement [59] and mindfulness [31], and at interventions for children with specific disabilities such as autism [28]. Most of these analyses have used quantitative data.

Nonetheless, researchers have found qualitative research methods to be suitable for in-depth investigation of an intervention’s active ingredients [29, 33, 34]. Interviewing parents on their recent experience with a DPT can shed light on which components or program characteristics enhanced their ability to learn and integrate effective parenting practices into their daily lives. The detailed descriptions provided by intervention users point to the effectiveness of specific components embedded in the intervention and how participants used these functionalities in practice. For example, digital triggers, such as text messages sent to users, are embedded in technology-based interventions to maintain the salience of specific therapeutic goals in the user’s mind [3, 18, 54–57]; however, it is important to consider how these triggers actually promote action (i.e., behavior change) in order to ensure that these active ingredients are being properly utilized. Such feedback could be collected directly from users engaged with the intervention, using qualitative methods. Users’ perceptions of program components they found helpful - collected after they have used the program for a specific clinical aim – complements user-centered design approaches for intervention’s development. In user-centered design carried out during program development, data about users’ needs is collected with the aim of designing a product (i.e., intervention) that better addresses user needs [70]. The examination of user perceptions about active ingredients they found helpful post use, complements the former activity by enabling scholars to identify ways to improve the intervention’s efficacy post deployment [71].

Studies on active ingredients using qualitative methods have been conducted on interventions focused on children with autism [29, 36], in-person parent training programs for toddlers and young children [20, 44, 51], and interventions for parents of children with diabetes [53]. However, to the best of our knowledge, none have focused on DPTs aimed at addressing children’s problem behaviors.

Our current study addresses this gap by exploring parents’ experiences of the active ingredients in two self-guided DPTs, tested within a randomized controlled trial (RCT) setting. This RCT examined the impact of incorporating design features aimed at helping parents adhere to the therapeutic process, as part of efforts to address the major challenge of improving user engagement to digital health interventions (e.g., Fleming et al., [26]. The DPTs implemented in this study shared similar evidence-based content but differed in terms of their functionality, as implemented through varying levels of therapeutic persuasiveness (TP). The term therapeutic persuasiveness (TP) was developed based on a comprehensive systematic review aimed at identifying all published quality criteria of eHealth interventions [10]. TP describes design features aimed at helping users make positive changes in their lives and includes elements such as ‘call to action’, monitoring and ongoing feedback, and program adaptations based on the user’s current state. Similar design features have been successfully incorporated to eHealth interventions [43, 72] and the quality of TP was identified in a previous study as the most robust predictor of user engagement in real-world usage [6, 8]. In the RCT described, the standard intervention (DPT-STD) included seven e-learning modules, while the other intervention (DPT-TP) incorporated similar content along with TP design features intended to encourage parents to make positive changes in their parenting behaviors by reaching out to them during concrete focusing phases within the program (see methods for a complete description). In this RCT, the researchers found that 68.9% of the DPT-TP parents completed all program modules, compared to only 27.9% of DPT-STD users [13]. Although both DPTs resulted in a significant improvement in children’s problem behaviors, the difference between both programs was significant and favored DPT-TP users.

Building on these results, the current study made use of qualitative data collected via interviews and parents’ responses to open-ended questions to identify the active ingredients noted by parents who used each DPT. We explored which components parents deemed important in general, and whether those using the DPT with enhanced functionality experienced different active ingredients or experienced the same ones but with a different magnitude of acknowledged support. We postulated that this qualitative study would yield new insights into the added value of these design features beyond the inherent value of evidence-based content.

## Methods

This study was follow-up research to an RCT conducted by Baumel and colleagues [13] that evaluated two DPTs for child problem behaviors that differed in their level of TP. The protocol was approved by the institutional review board of University of Haifa (approval number: 058/22).

### Participants and Recruitment Procedure

Parents were invited to join the study via a campaign on Facebook between May and July 2022. Those who submitted their contact information were then prompted to complete a short eligibility screening questionnaire that covered both inclusion and exclusion criteria, as well as questions regarding the behavior of their child.

The criteria to qualify for the study included: (1) being the parent of a child aged 3 to 7 who exhibited (2) elevated levels of problem behaviors based on the Eyberg Child Behavior Inventory (ECBI) subscales (ECBI-problem ≥ 15 or ECBI-intensity ≥ 132), and (3) possessed a smartphone with both cellular and internet connectivity. The exclusion criteria were: (1) the child was already receiving treatment for behavioral or emotional issues, or the parent was involved in a different parent training program; and (2) the child had a diagnosis of intellectual disability or developmental delay.

Prospective parents were contacted by phone to confirm the criteria from their initial screening and to provide them with more details about the program and the study. Those interested in participating were then guided to electronically sign a consent form and complete the baseline assessment. Eligible parents were then randomly assigned to one of the two DPTs and given the login information through an email and a text message. Parents whose children did not meet the study criteria received information about available public mental health services.

### Overview of Interventions

The DPTs utilized in this study operate on the principle that parents’ actions and responses can significantly shape their child’s behavior [25, 62]. The DPT was based on Prof. Baumel’s protocol, integrating proven strategies from renowned parent training programs, including Helping the Non-Compliant Child, Incredible Years, Strongest Families, and Triple P. The protocol includes seven modules, each focusing on a specific theme: (1) introduction to parent training; (2) positive interactions and quality time; (3) parental emotion regulation; (4) effective routines and clear ground rules; (5) recognizing positive behaviors and ignoring minor negative behaviors; (6) overcoming disobedience; and (7) mindful parenting and communication between partners [13]. Two versions of the program were used:

#### Standard DPT (DPT-STD)

This version encompassed all seven modules mentioned above. Each module utilized texts, videos, and interactive features (e.g., multiple-choice questions with direct feedback), and required between 10 and 25 min to complete.

#### DPT with enhanced TP quality (DPT-TP)

This version incorporated the DPT-STD content along with additional TP features designed to enhance the salience of the therapeutic activities in the parent’s mind. In this version of the program, parents went through a learning phase, followed by a 1–2 week focusing phase that incorporated the following design features:

1. Call to action: Each day parents received timely text messages with tips or motivating messages based on their current module [55].
2. Monitoring and ongoing feedback of user state: Each day, parents were invited to answer a short questionnaire focusing on the current module’s therapeutic activities. The repetitive nature of the questions was designed to enhance the salience of newly acquired skills [7]. Based on their answers, parents received automated feedback with additional information and ideas. More detailed weekly feedback was also provided.
3. Adaptation to user state: Each parent was presented with a personal program plan based on a short intake questionnaire. The personal programs varied in their modules – i.e., out of the seven modules available, four were mandatory and three were optional – and the order in which the modules were presented to the parent.

To maintain content consistency across both interventions, we examined all unique content in the DPT-TP focusing phase, such as tips and new suggestions, and integrated it into the DPT-STD learning modules.

## Measures

Data for this study were collected through semi-structured interviews, conducted via Zoom, and written answers to open-ended questions that were part of the post-intervention measurements, administered via Qualtrics.

### Semi-Structured Interview

The questions for this interview were designed to capture the unique experience of parents engaging with the program while managing their daily work and family schedules. Parents were prompted to elaborate on their responses, share examples, or relate to program features relevant to their answer. The questions were as follows:

1. Have you found yourself thinking about the skills or tools presented in the program while spending time with your child, such as during the afternoon or evening? Please share more about your experience.
2. When you are with your child, for example, in the afternoon, how clear is it to you how to address or react to your child’s behavior?
3. How clear is the connection between the goals you had when joining the program and the skills and tools you acquired through the program?
4. Did you feel that the program motivated you to change your parental behavior? If so, in what ways?
5. Have you noticed a change in your availability or presence with your child since participating in the program? Please elaborate.

#### Open-ended questions

These questions were included in the post-intervention assessment, which was administered via Qualtrics. Parents were invited to respond in writing to the following questions, with no restriction on the length of their answer: What advantages, if any, did you find in using the program to create a change in the way you react to your child? Did the program assist you in creating a positive change with your child? What aspects of the program did you appreciate the most?

### Data Collection and Analysis

Participation in the RCT involved completing the post-intervention assessment, which included the open-ended questions. We contacted parents who participated in the RCT and invited them via email to participate in an interview conducted via Zoom. The interviews were held until data saturation was reached for both treatment conditions, meaning that no additional unique content was identified [61]. We then conducted a thematic analysis of parents’ interview responses according to the six steps suggested by Braun and Clarke [16]. The first author transcribed the interviews, looked for initial codes, and searched for initial themes. Subsequently, CRS and AB, in an iterative process, defined and refined the final themes. This analysis of in-depth interviews enabled us to build a theme map of the active ingredients identified by parents who had engaged with the program.

In the second step, we collected responses to the open-ended questions included in the post-intervention assessment. For this analysis, 31 participants from the DPT-STD group and 33 participants from the DPT-TP group provided usable data; the others did not provide detailed responses (i.e., they stated that the program had helped them to make changes at home, but they did not elaborate further). We analyzed the responses to these questions in light of the themes derived in the first step. This process enabled us to estimate the acknowledged importance of each theme, or active ingredient, for a larger sample of parents who had engaged with the program. Since responses to open-ended questions are typically less detailed than interview answers, they may not have encompassed all the active ingredients that parents found helpful during the intervention. However, the extent to which themes were presented in parents’ answers did allow us to capture the ingredients that were most salient in parents’ minds when responding to the questions. At this stage, we reviewed each open-ended response, identifying content related to one of the five main themes derived in the first step. We then tallied the number of parents relating to each theme and further calculated the percentage of parents relating to each theme out of the total number of parents who responded to the open-ended questions.

## Results

Sixteen parents of children with problem behaviors participated in the semi-structured interviews, and the responses to open-ended questions from 64 parents were analyzed. Table 1 presents demographics for all participant groups. There were no significant differences between DPT-STD and DPT-TP conditions in terms of group characteristics.

**Table 1:** Participant demographic characteristics by intervention condition

		Interview participants				Open-ended question participants			
		**DPT-STD** (*n*** = 8)**	**DPT-TP** (*n*** = 8)**	**Mann-Whitney U**	***p***	**DTP-STD** (*n*** = 31)**	**DPT-TP** (*n*** = 33)**	t (62)	***p***
**Continuous**		**M (SD)**	**M (SD)**			**M (SD)**	**M (SD)**		
Parent age (years)		38.63 (5.88)	35.63 (3.16)	24.00	0.442	36.42 (3.70)	36.15 (3.34)	0.30	0.76
Child age		5.31 (1.58)	4.61 (1.08)	23.5	0.382	5.13 (1.45)	4.88 (1.26)	0.77	0.45
Number of children in the family		2.50 (0.53)	2.38 (0.74)	26	0.574	2.52 (0.724)	2.76 (1.03)	-1.09	0.27
**Categorical**		**N (%)**	**N (%)**	**χ** ^2^	*p*	**N (%)**	**N (%)**	**χ**^2^(df)	***p***
Leading parent gender	Male	0 (0%)	1 (14.23%)	1.07	0.30	1 (3.2%)	2 (6.1%)	0.29	0.59
	Female	8 (100%)	7 (85.71%)			30 (96.8%)	31 (93.9%)		
Child gender	Male	6 (75%)	5 (62.5%)	0.29	0.59	18 (58.1%)	19 (57.6%)	0.002	0.97
	Female	2 (25%)	3 (37.5%)			13 (41.9%)	14 (42.2%)		
Participating	Both parents	6 (75%)	5 (62.5%)	0.29	0.59	17 (54.8%)	22 (66.7%)	0.94	0.33
	One parent	2 (25%)	3 (37.5%)			14 (45.2%)	11 (33.3%)		
Education^a^	High school	0	0	-	-	4 (12.9%)	3 (9.1%)	0.24	0.62
	Above	8 (100%)	8 (100%)			27 (87.1%)	30 (90.9%)		
Household income^b^	< 15 K	1 (12.5%)	0	2.82	0.24	5 (16.1%)	6 (18.2%)	0.58	0.75
	15–18 K	3 (37.5%))	1 (12.5%)			9 (29.0%)	12 (36.4%)		
	> 18 K	4 (50%)	7 (87.5%)			17 (54.8%)	15 (45.5%)		
Religiosity	Secular	5 (62.5%)	5 (62.5%)	3.00	0.23	17 (54.8%)	18 (54.5%)	0.42	0.24
	Traditional	3 (37.5%)	1 (12.5%)			11 (35.5%)	8 (24.2%)		
	Religious	0	2 (25/%)			3 (9.7%)	7 (21.2%)		
Hours of work/study per week^a^	> 10 h	1 (12.5%)	0	3.08	0.215	4 (12.9%)	5 (15.2%)	1.14	0.57
	10–29 h	0	2 (25%)			3 (9.7%)	6 (18.2%)		
	> 30	7 (87.5%)	6 (75%)			24 (77.4%)	22 (66.7%)		

^a^ Refers to the parent leading the intervention

^b^ In Israeli shekels (ILS)

### Thematic Analysis of Interview Responses

**Fig. 1:**
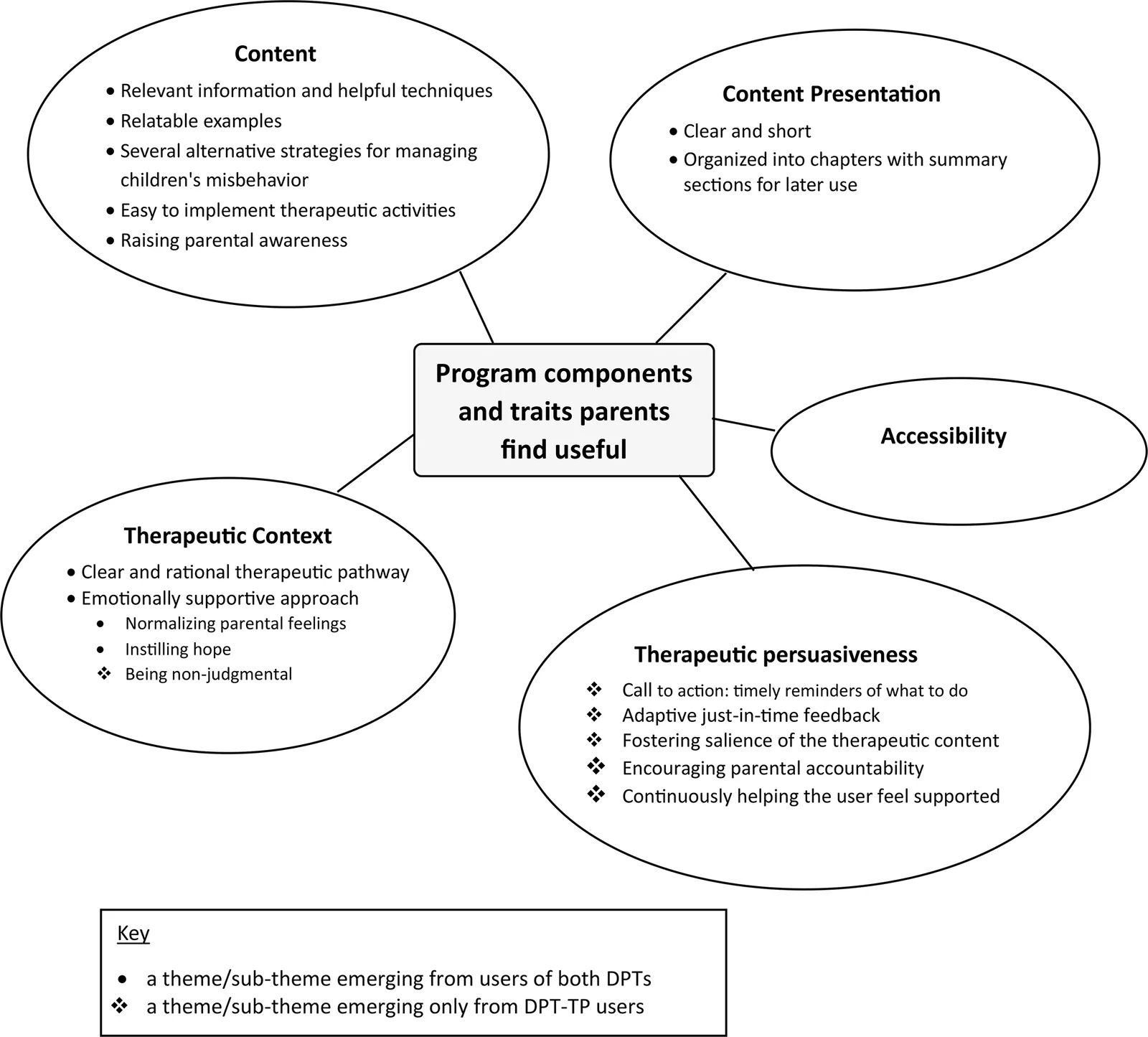
Map of themes and sub-themes

Through the thematic analysis, the data was organized around five themes: (a) content; (b) content presentation; (c) accessibility; (d) therapeutic context; and (e) therapeutic persuasiveness (see Fig. 1 for a representation of themes and their sub-themes). Notably, themes (a) to (c) emerged in the responses of parents in both programs (DPT-STD and DPT-TP), whereas one sub-theme in theme (d) and theme (e) emerged only in the responses of DPT-TP parents.

In this section, we present a short description of each theme and sub-theme, with sample quotes from parents interviewed in the studies.

### First Theme: Content

This theme relates to the benefits that parents identified in the program’s content, i.e., information, and techniques taught, and how these benefits helped to facilitate the desired change in their child. It encompasses five sub-themes concerning the content itself and the cognitive changes parents experienced while interacting with the program.

#### Sub-theme 1 – Relevant Information and Helpful Techniques

This sub-theme relates to parents’ view of the content itself, that is, the psychoeducational knowledge and tools provided. Parents reported that the content was practical, and relevant to their child’s behavior problems; implementing the efficient tools and knowledge promoted a positive change at home. For instance, one parent replied, “We always give positive feedback to our child… but here we got some new ideas, and we implemented them”.

#### Sub-theme 2 – Relatable Examples

This sub-theme represents parents’ recognition that the examples and case studies given were relatable and specific, detailing exactly what could be said and done in different situations. For example, one parent noted, “If my child starts to hit… it was clear, you know exactly what you have to do!”.

#### Sub-theme 3 – Several Alternative Strategies for Managing Children’s Misbehavior

The third sub-theme relates to parents’ appreciation of the variety of strategies and ideas given in each section. For example, one mother shared, “We were advised to try and see what works for us. There were a variety of options to choose from… different children react differently [to the same approach, CRS]”.

#### Sub-theme 4 – Easy to Implement Therapeutic Activities

The fourth sub-theme pertains to parents’ perception of the therapeutic activities and examples as manageable and feasible to implement. For instance, one mother stated, “For every example provided, I thought to myself, well, this isn’t too difficult. I can manage this”.

#### Sub-theme 5 – Raising Parental Awareness

The last sub-theme relates to the insights and reflections that parents had about themselves and their child’s behavior, which emerged during or after their engagement with the content. According to the parents interviewed, these cognitive changes broadened their understanding and enhanced their ability to plan and manage their responses to their child. We identified four domains where these reflections influenced parents’ perceptions: the first one related to thoughts concerning future responses to their child (e.g., “While reading, I took time to think things over… I thought and I planned my reactions”). The second related to the collaborative processes between both parents, leading to joint decisions on how to respond to their child (e.g., “At our home, we initiated a discussion about these topics, which we usually don’t do. Each of us shared his thoughts and the actions he thought we should take”). The third refers to insights described by parents regarding the impact of their reactions on their child’s behavior, which led to changes in their parenting practices (e.g., “The program taught us that if we change our interpretation of situations, then our child’s reaction will also change”). The last concerns parents’ perception of regaining their ability to pause and plan their reactions to their child, rather than acting impulsively. A mother shared, “The program raised my awareness that things don’t have to be automatic… I don’t have to be responsive all the time. I can plan ahead”.

### Second Theme: Content Presentation

This theme captures parents’ perceptions of the way the content was designed and organized, and how these aspects aided them in understanding and retaining the knowledge presented. Two sub-themes emerged from the interviews:

#### Sub-theme 1 – Clear and Short

Parents appreciated the concise language used, which made it easier for them to consume the content. For example, a mother replied, “The wording was very very short and to the point, there wasn’t too much to read”.

#### Sub-theme 2 – Organized into Chapters with Summary Sections for Later use

This sub-theme relates to the organization of the content into chapters with a summary at the end of each chapter, which could be downloaded and printed. According to the parents, these features facilitated their ability to revisit the program and find pertinent information when needed. A mother shared, “The program being organized into chapters was the best thing for me as a parent. I could go back to the relevant chapter, look at the tools mentioned, and see how I should address my child now”.

### Third Theme: Accessibility

This theme refers to the core strength of online interventions, their accessibility, which enables parents to conveniently engage with the program at home, in parallel with their work and home schedules. Parents appreciated not having to make the effort to attend face-to-face consultations and being able to revisit the program’s content at their own convenience. For instance, a mother of four young children with a full-time job shared, “I didn’t have to find a babysitter for my four kids. The intervention was with me all the time, on my cellphone”. Another mother stated, “The fact that I could revisit the content again and again was very convenient… I could do it at my own pace”.

### Fourth Theme: Therapeutic Context

This theme encompasses parents’ perceptions that the program delineated a clear therapeutic path, guiding them through essential knowledge and suggested activities, while maintaining an emotionally supportive environment. These features may help parents adhere to the program, especially when the intervention is fully self-guided and does not offer human support [10].

#### Sub-theme 1 – Clear and Rational Therapeutic Pathway

This sub-theme relates to the program’s perceived ability to create and maintain a robust therapeutic pathway linking parental goals to the therapeutic activities offered by the program. For example, a participant shared, “It was clear that the intervention is build step-by-step… you need to have some background knowledge”. Another participant stated, “It was explained why I should change the way I react to my child… and how these changes would affect both my child and my relationship with him. The explanations made sense and were convincing”.

#### Sub-theme 2 – Emotionally Supportive Approach

This sub-theme encompasses three narratives shared by parents, all reflecting their perceptions of the program as being warm, supportive, empathetic, and encouraging. The first narrative refers to parents’ perceptions that the program normalized the difficulties they were experiencing with their child, showing them that these challenges were common among families with young children. For instance, one mother shared that the program had made her feel that things are “normal… It’s not only me in this hell, but it is something that is known and common”. The second narrative relates to parents’ feelings of hope, evoked by the program, that there are steps they can take to improve their child’s behavior and their relationship. For example, a mother shared that engaging with the program had given her the feeling that “today things didn’t work out. And it might be the same tomorrow. But eventually – things will improve”. The last narrative emerged in the responses of DPT-TP users only and reflects parents’ feelings that their behavior was acceptable, and they could share their true experiences with their child without facing criticism (e.g., while answering the daily questionnaire). A mother shared, “I felt no criticism or judgment. It gave me the legitimacy to be real. I didn’t feel I had to lie or pretend”.

### Fifth Theme: Therapeutic Persuasiveness

This theme refers to the implementation of persuasive design features in the program. Parents identified these program components as being helpful in supporting them to engage in therapeutic activities and ultimately facilitating changes in their behavior. This theme emerged in the responses of parents in the DPT-TP group only.

#### Sub-theme 1 – Call to Action: Timely Reminders of what to do

This sub-theme relates to parents’ reflections on the helpfulness of the daily SMS reminders, which encouraged them to take action and complete the therapeutic activities recommended by the program. For example, a mother shared, “This tiny reminder… just before picking up my child from kindergarten, helping me remember what I should do”.

#### Sub-theme 2 – Adaptive just-in-time Feedback

This sub-theme relates to parents’ appreciation of receiving feedback and guidance from the program exactly when they needed it – following the daily questionnaire. Based on their reports, the program would suggest additional relevant information and make further recommendations. As one mother reflected, “The fact that after filling out the daily questionnaire, you provided us with additional information… an extra reading material if something didn’t work out for us”.

#### Sub-theme 3 – Fostering Salience of the Therapeutic Content

This sub-theme encompasses parents’ reflections on two program components designed to reach parents in their daily environment and keep the program’s content salient in their mind: the daily SMS triggers and the daily questionnaires. A parent concluded, “I received daily text messages with tips, and every night I filled out the questionnaire. It simply helped me keep things in mind… this was the key, the meaningful aspect”.

#### Sub-theme 4 – Encouraging Parental Accountability

This sub-theme encompasses parents’ reports on the impact of the daily SMS messages and questionnaires in encouraging them to actively engage with the program and apply the newly acquired tools with their children. Being actively engaged with the program and executing the therapeutic activities recommended for each chapter gradually shifted the responsibility back to the parents, making them the owners of the therapeutic process. For example, a parent shared, “The program brings back responsibility and control to me”. Another parent stated that she and her spouse “made an effort to implement as many of them (i.e., tips and tools, CRS) as possible and regularly evaluated what we got right and what we got wrong”.

#### Sub-theme 5 – Continuously Helping the user feel Supported

This sub-theme concerns parents’ perception of the program providing them with support and accompaniment throughout the therapeutic process, facilitated by the daily SMS messages and questionnaires. For example, a mother shared, “The daily text messages were something I looked forward to… You became an integral part of my daily routine… I felt you were with me all the time, accompanying me… I was sorry when the program was over. Could you stay a little longer?” A different mother anthropomorphized the program, stating, “When I received the SMS messages, I felt that I was not alone. There was someone with me, someone who remembers me… like a big brother accompanying me”.

### Analysis of Responses to open-ended Questions

We analyzed 31 responses from DPT-STD users and 33 responses from DPT-TP users. The results, presented in Table 2, show that all DPT-STD users mentioned the program’s content when asked to specify program components that had helped them to create a positive change with their child. Approximately one-third of the parents related to the content presentation, a quarter to program’s accessibility, and only one parent mentioned the program’s perceived ability to foster a therapeutic context. By contrast, the findings from DPT-TP users present a different picture: fewer parents mentioned the program’s content, content presentation, and accessibility (84.85%, 24.24% and 12.12%, respectively), while 36.36% of the parents highlighted the therapeutic persuasiveness quality of the program as the key factor that improved their capacity to facilitate change. Chi-square tests revealed no significant associations between parents’ demographics (income level, religiosity, and number of parents participated in the intervention) and their tendency to describe a specific theme (χ²_s_ ≤ 2.28, and p _s_ ≥ 0.14).

**Table 2:** Results from open-ended questions: number of parents relating to each main theme, according to program version (% out of total number of parents)

	Content	Content presentation	Accessibility	Therapeutic context	Therapeutic persuasiveness
DPT-STD (*n* = 31)	31 (100%)	10 (32.26%)	8 (25.81%)	1 (3.23%)	0 (0%)
DPT-TP (*n* = 33)	28 (84.85%)	8 (24.24%)	4 (12.12%)	1 (3.23%)	12 (36.36%)

## Discussion

This study extends research on the active ingredients of DPTs by examining how parents experience the support they receive from self-guided DPTs. Parents from both intervention groups found the content, content presentation, accessibility, and maintenance of the therapeutic context to be helpful during their process of change. Users of the enhanced-TP program also emphasized TP components that encouraged parental adherence to therapeutic activities, e.g.,‘call to action’ text triggers and adaptive feedback. According to the parents, these features nurtured the salience of the desired activities above competition, encouraged a parental sense of responsibility, and provided a supportive context. Parents in the larger sample also recognized the same aspects of the program. All users of DPT-STD described the content as being beneficial to their process of change; among DPT-TP users, 84.85% also mentioned the content, but 36.36% of them described the TP qualities of the DPT as being helpful for creating positive change with their child.

The study’s findings align with research underscoring the significance of content and accessibility in eHealth interventions as effective active ingredients [19, 34, 47]. Alongside content and accessibility, recent studies have also highlighted the importance of tailoring and personalizing eHealth interventions to improve engagement and outcomes [16, 18, 49, 58], which aligns with our findings as well. Three key functionalities were identified by the parents and attributed to the tailored digital triggers (i.e., timely text messages) and the daily questionnaire with personal feedback. First, fostering content salience was achieved through ‘call to action’ messages sent in the afternoon, prompting desired actions in the parents’ natural environment [30, 60], as well as through the daily questionnaire sent in the evening. These reminders of therapeutic goals delivered twice daily, along with personalized information, were reported by parents as helpful for staying focused on the process, while overcoming competing cognitive, emotional, or environmental demands [7, 65]. Second, fostering parental accountability was highlighted. Accountability relies on the anticipation of an interaction between the user and the program (i.e., the daily questionnaire prompt), creating a sense that the users will be held accountable for their behavior [17], a perception that may boost adherence to the therapeutic process. Third, parents reported a sense of being supported throughout the therapeutic process. This perception may be linked to the regularly sent messages (i.e. daily text messages and questionnaire prompts) which evoke a social response towards the DPT, similar to how parents might interact with another person - an effect previously demonstrated in digital interventions [18].

The uniqueness of this study, comparing two DPTs within an RCT, enables us to understand the specific added value of TP functionality. The qualitative findings, along with the quantitative results from the RCT on these two DPTs [13], support TP as a means to enhance engagement and user outcomes in eHealth interventions, given that the level of TP was the sole difference between the two DPTs. While the quantitative results highlight TP’s effectiveness in boosting adherence to the intervention and outcomes, the qualitative results provide insights into how users experience these features in their home environment and aid in the formulation of hypotheses about the ways in which parents change their behavior while interacting with interventions. These insights are essential for improving eHealth interventions and mitigating the high dropout rate reported in the literature [4, 18, 35].

For example, parents who received the DPT-TP reported that completing a short daily questionnaire and receiving immediate feedback was helpful for maintaining the salience of the themes being learned and provided a platform for reflecting on their parenting practices and their impact on their child. These activities may have enhanced their parental skills and self-efficacy, ultimately leading to improved child behavior. Future research could further investigate these notions using mediation analysis [23, 42, 67].

The beneficial features identified by parents as helpful could be incorporated into protocols that blend in-person and digital tools within parent training programs. In particular, blended interventions could integrate eHealth features between traditional therapy sessions [2], including automatic reminders of targeted activities, or enabling therapists to monitor patient’s progress and provide ongoing feedback between successive sessions. Preliminary studies in the area of child problem behaviors [38] and depression [39, 63] have yielded promising results for such blended care. Further research should focus on enhancing the effectiveness of traditional behavioral parent training by incorporating digital components that assist parents in achieving their therapeutic goals between sessions.

Utilizing the benefits of the specific TP features recognized as helpful by parents, may enhance accessibility of evidence-based DPTs for high-needs families [68]. Since these features are designed to help parents minimize the effort needed to integrate and practice the therapeutic activities while already engaging with their children, they can mitigate the need for substantial resource investment [7]. Future qualitative data collected from these families could help identify the active ingredients they found most helpful and uncover any unique components valued by these populations.

Another possible direction for future research is to examine the similarities and differences between active ingredients, as reported by users of self-guided versus guided DPT-TP versions. Although recent meta-analyses on eHealth interventions suggest that the level of support does not influence outcomes [14, 21, 66], other studies indicate that eHealth interventions with human support yield better outcomes [11, 24, 32, 48]. Qualitative data from such studies could lead to a better understanding of the functionality of human support, as well as to how TP functionality might mimic human support. These insights could serve to improve parental engagement and outcomes for both parents and children.

### Limitations

As with all qualitative research, this study has several limitations. First, there is a potential selection bias, resulting in a cohort of parents who found the DPT helpful for their child’s problem behaviors and were therefore more motivated to participate in the interview. In addition, a recall bias might be present, as parents relied on their memories after the intervention had already ended. These biases may affect our ability to generalize the results. To mitigate these limitations, we supplemented our analysis with responses to open-ended questions included in the post-intervention assessment for all parents. Since these responses corroborated the aforementioned themes, the risk from these biases is estimated to be low. Lastly, this study focuses on the active ingredients that parents found helpful in facilitating behavior change. Therefore, we did not ask parents about components they found unhelpful or that were absent from the DPTs used in this study. These aspects could be explored in future usability studies.

## Summary

The success of behavioral interventions largely depends on parents’ willingness to actively engage and integrate new behavioral techniques, which can lead to positive change [22]. In the realm of eHealth interventions, focusing on beneficial product design is imperative to sustain parental engagement and enhance a program’s efficacy. One method of achieving this goal is through the collection of qualitative feedback from program users. This approach has proven effective in identifying the active ingredients that facilitate the process of change, and thus it can serve as a valuable input for the design process.

This study demonstrates that parents using interventions with different design features perceive different active ingredients, despite the content of the interventions being similar. This observation underscores the importance of adequate product design to support the desired therapeutic change, since active ingredients are used to direct the user’s behavior [35]. Further studies on the interaction between active ingredients, user experience, and outcomes could enhance our knowledge of the theoretical and empirical aspects of DPTs.

## Data Availability

No datasets were generated or analysed during the current study.
